# Study on the Preparation and Properties of Vegetation Lightweight Porous Concrete

**DOI:** 10.3390/ma17010251

**Published:** 2024-01-03

**Authors:** Qingyu Cao, Juncheng Zhou, Weiting Xu, Xiongzhou Yuan

**Affiliations:** 1 MMC Group, Central Research Institute of Building and Construction, Beijing 10088, China; achero@126.com; 2 School of Civil Engineering, Inner Mongolia University of Science and Technology, Baotou 014010, China; 17692455153@163.com; 3 School of Materials Science and Engineering, South China University of Technology, Guangzhou 510641, China; 4 School of Traffic and Environment, Shenzhen Institute of Information Technology, Shenzhen 518172, China

**Keywords:** porosity, mechanical property, alkalinity, permeability coefficient

## Abstract

The objective of this study is to formulate vegetated light porous concrete (VLPC) through the utilization of various cementing materials, the design of porosity, and the incorporation of mineral additives. Subsequently, the study aims to assess and analyze key properties, including the bulk density, permeability coefficient, mechanical characteristics, and alkalinity. The findings indicate a linear decrease in the volume weight of VLPC as the designed porosity increases. While higher design porosity elevates the permeability coefficient, the measured effective porosity closely aligns with the design values. The examined VLPC exhibits a peak compressive strength of 17.7 MPa and a maximum bending strength of 2.1 MPa after 28 days. Notably, an escalation in porosity corresponds to a decrease in both the compressive and the bending strength of VLPC. Introducing mineral additives, particularly silicon powder, is shown to be effective in enhancing the strength of VLPC. Furthermore, substituting slag sulfonate cement for ordinary cement significantly diminishes the alkalinity of VLPC, resulting in a pH below 8.5 at 28 days. Mineral additives also contribute to a reduction in the pH of concrete. Among them, silica fume, fly ash, fly ash + slag powder, and slag powder exhibit a progressively enhanced alkaline reduction effect.

## 1. Introduction

In recent years, vegetation lightweight porous concrete (VLPC) has emerged as an innovative integration of traditional concrete technology with the advantages of horticulture [1,2]. VLPC boasts a considerable pore volume and a distinctive pore structure [3,4,5,6], resulting in enhanced water permeability [7], efficient heat dissipation capacity [8], and notable sound absorption capability [9]. These inherent characteristics render VLPC highly valuable for mitigating urban waterlogging [10], minimizing noise pollution [11], and fostering urban ecological development. Consequently, VLPC plays a pivotal role in the implementation of the concept of sponge cities [3]. By integrating plants within a porous concrete medium, VLPC enables the unhindered movement of water, air, soil, and roots. This environmentally friendly material finds frequent application in valley slopes and hilly areas, where it serves to enhance landscaping and to mitigate the risk of slope landslides. Nevertheless, in practical applications, the predominant cement used is typically ordinary Portland cement. This type of cement generates a substantial amount of the hydration product Ca(OH)_2_ during the hydration process, creating an alkaline environment that is unfavorable for vegetation growth [12,13]. The optimal pH range for plant growth is between 8 and 10, whereas the pH value of ordinary concrete is approximately 13—clearly unsuitable for the formulation of VLPC [14].

Numerous researchers have undertaken comprehensive studies with the objective of creating an internal environment within VLPC that promotes vegetation growth [15,16,17,18]. Li et al. [19] observed that, at a water–cement ratio of 0.26 and a targeted porosity of 26%, both the pH value of the soil and the concrete porosity reached their lowest levels. This condition was identified as providing the most optimal environment for vegetation growth. These findings underscore the significance of considering the porosity and pH value as critical factors influencing the growth and development of vegetation in VLPC. Peng et al. [20] conducted a comparative analysis of the permeability of concrete incorporating fly ash and silica fume. Their findings revealed that the addition of fly ash or silica fume led to a reduction in permeability while concurrently improving the strength of the concrete. Consequently, the determination of the optimal quantity of fly ash or silica fume in the manufacturing process of VLPC should be guided by primary performance criteria. Additionally, some scholars have noted that altering the raw material composition has a positive impact on enhancing the alkaline environment within the concrete. Yang et al. [21] investigated the evolution process and mechanism of the engineering performance of vegetated concrete under atmospheric freeze–thaw (F-T) test conditions. The findings indicated a decline in the acoustic wave velocity and cohesive forces, coupled with an increase in the permeability coefficient of vegetated concrete due to the effects of F-T action. The reduction in cohesive force was closely linked to an overall decrease in the content of the gelling hydration products in the vegetated concrete. Additionally, the diminished nutrient retention capacity of vegetated concrete was primarily associated with the disintegration and fragmentation of larger aggregates induced by F-T action. Cheng et al. [13] devised 10 sets of porous concrete specimens using mixed ratio columns to examine the influence of various parameters, including fly ash and silica fume contents, on the mechanical properties, porosity, and pH value of porous concrete. The outcomes of the study revealed that the compressive strength and porosity of the porous concrete, measured after 28 days, were at least 13 MPa and 21%, respectively. This suggests that the porous concrete exhibits suitability for slope protection in practical engineering applications. Additionally, all grass species thrived in the eco-modified soil and porous concrete environment throughout the observation period, achieving a vegetation coverage rate of 96% or higher. It was also observed that the root system permeated the pores of the porous concrete, reaching the bottom after 8 weeks. Kim et al. [22] conducted a study evaluating the vegetation growth performance of porous vegetated lightweight porous concrete (VLPC) blocks prepared with blast furnace slag cement. After placing the test blocks in the field, plant growth was monitored, and seeds began to germinate a week after sowing. Remarkably, after 6 weeks, the plant length exceeded 300 mm, and the average coverage reached an impressive 90%. These findings unequivocally demonstrate that VLPC, prepared with blast furnace slag cement, is well-suited for environmental remediation projects. Moreover, the selection of aggregates and cement substitutes was identified as a significant factor influencing the vegetation growth performance of VLPC. Wang et al. [1] found that soaking in ferrous sulfate solution for 6 days, oxalic acid solution for 10 days, and water for 26 days can reduce the pH value of the internal pores of concrete to about 8, indicating that the alkali reduction effect can be achieved in a short time by using an alkali reduction solution, and the other properties of vegetation concrete are not significantly affected. Bao et al. [8] pioneered the development and investigation of vegetation pervious concrete with low alkalinity. The reduction in concrete alkalinity was achieved through the addition of admixtures to the cement slurry. The study utilized XRD (X-ray diffraction) and compression tests to examine the impact of the admixture content on the concrete basicity and compressive strength. The results indicated a decrease in the alkalinity of the cement samples with increase in the admixture content and successful vegetation growth on the pervious concrete. Moreover, by elevating the admixture content to approximately 3.6%, the compressive strength of the pervious concrete exceeded 25 MPa.

Building upon these findings, the present study undertook the preparation of various types of plant-growing lightweight porous concrete. To cater to the requirements of vegetation growth, essential performance indicators, including the bulk density, strength, alkalinity, porosity, and water permeability coefficient, were meticulously examined. The objective was to furnish a scientific foundation and technical support for the application of this material in construction engineering, urban greening, as well as energy-saving and environmental protection initiatives.

## 2. Materials and Methods

### 2.1. Materials

P.II 52.5 grade ordinary Portland cement (OPC) (Onoda Cement Factory, Dalian, China), meeting the Chinese standard GB/T 175-2007 [23], and 52.5 grade slag sulphoaluminate cement (SSC) (Polar bear Co., Tangshan, China), meeting the Chinese standard GB/T 20472-2006 [24], were chosen for the experiment. The specific surface areas of these cements were 392 m^2^/kg and 450 m^2^/kg, respectively. The coarse aggregate utilized was shale ceramic granule 900 grade lightweight aggregate, with a density of 960 kg/m^3^ and a particle size ranging from 10 to 15 mm. Fly ash (Datang Tongzhou thermal power plant, Tangshan, China) was employed as low-calcium work-grade fly ash (FA) with a density of 2.13 g/cm^3^, a fineness of 6% (45 μm sieve-screened), and a water demand ratio of 0.95. The slag powder used was S95 granulated blast furnace slag (GBFS) (Douhe power plant, Tangshan, China), characterized by a density of 2.86 g/cm^3^, a specific surface area of 590 m^2^/kg, a mobility ratio of 97, and a 28-day activity index of 104. The chemical additive chosen was JM-2 polycarboxylic (Sobute New Materials Co., Nanjing, Jiangsu, China) acid water-reducing agent, achieving a water reduction rate of 36.7%. The chemical compositions of the cement, FA, GBFS, and silica fume (SF) (Elkem Materials Co., Guangzhou, China) are detailed in Table 1.

### 2.2. Mix Design

The preparation of vegetative lightweight porous concrete relies on effectively enveloping the coarse aggregate with cementitious material to create uninterrupted voids. However, conventional concrete proportion design methods do not meet the necessary criteria for void formation in this specific type of concrete. Therefore, it is essential to devise novel and effective design principles and techniques for the proportioning of lightweight and porous vegetative concrete.

The mix ratio of vegetated lightweight porous concrete is influenced by several crucial factors, including the water–cement ratio (R_W/C_), the design porosity (R_void_), the density (*ρ*), and the aggregate porosity (ν_c_). The specific calculations can be broken down into the following steps:

(1) Calculation of the amount of coarse aggregate per unit cubic meter.

(1) $$W_{G}=\alpha \cdot \rho_{G}\;(kg/m^{3}),$$

where α represents the correction coefficient, set at 0.98; $\rho_{G}$ signifies the compact packing density of coarse aggregate, measured in kilograms per cubic meter (kg/m^3^); and $W_{G}$ denotes the quantity of coarse aggregate per unit cubic meter, expressed in kilograms per cubic meter (kg/m^3^).

(2) Calculation of slurry volume of cementitious materials.

(2) $$V_{P}=1000-\alpha \cdot 10\cdot \left(100-\nu_{c}\right)-R_{void}\cdot 10\;(L/m^{3}),$$

where $V_{P}$ denotes the volume of cementitious material (kg/m^3^); $\nu_{c}$ denotes the dense pile porosity of coarse aggregate (%); and $R_{void}$ denotes the design porosity (%).

(3) Calculation of cement and water consumption per cubic meter.

(3) $$W_{C}=\frac{V_{p}}{R_{W/C}+\frac{1}{\rho_{C}}}$$

(4) $$W_{W}=W_{C}\cdot R_{W/C},$$

where $W_{W}$ denotes the amount of cement per unit cubic metre (kg/m^3^); $R_{W/C}$ denotes the water-cement ratio; $\rho_{C}$ denotes the density of cement (kg/m^3^); and $W_{W}$ denotes the amount of water used per unit cubic metre (kg/m^3^).

When incorporating fly ash, slag powder, silica fume, and other mineral admixtures, the dosage calculations are based on volume conversion corresponding to the volume of the cementitious material slurry. This involves following the aforementioned steps to calculate the dosage for each admixture. Typically, when the admixture dosage is small, its volume is not factored into the total volume of the slurry. The specific calculations for various groups are detailed in Table 2.

### 2.3. Specimen Preparation and Maintenance

**Figure 1:**
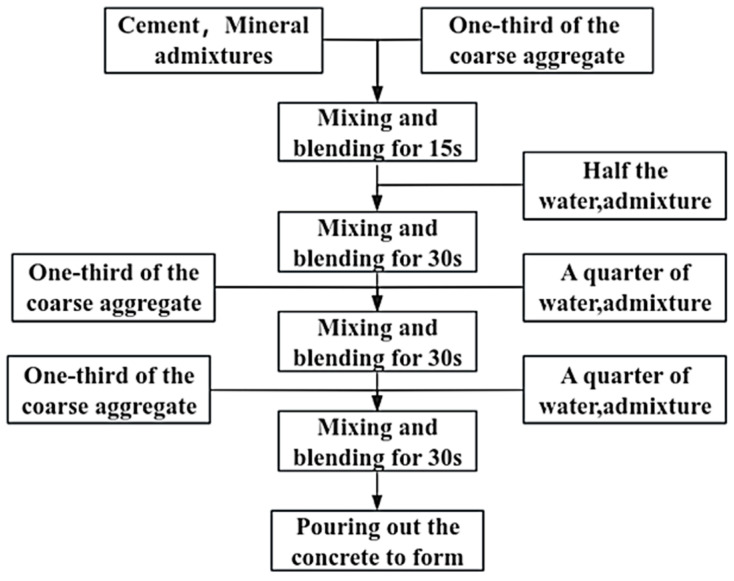
VLPC mixing process.

In contrast to ordinary concrete, the fluidity of VLPC is significantly reduced, attributed to its lower cement content, absence of fine aggregate, and the presence of a substantial amount of coarse aggregate. The internal friction between the slurry and coarse aggregate, along with the coarse aggregate itself, is notably high. This results in the slurry being prone to forming clusters, making it not only challenging to mix but also difficult to establish a continuous and cohesive structure. To ensure the comprehensive and effective coverage of the coarse aggregate surface by the cementing material slurry, a novel stirring process is introduced in the test, as illustrated in Figure 1. The mixing process involves several steps: initially, cement, mineral admixture, and one-third of the coarse aggregate are mixed and stirred for 15 s. Subsequently, half of the water and admixture are added, and the mixture is stirred for 30 s. Following this, a third of the coarse aggregate, along with a quarter of the water and admixture, is introduced into the concrete, and mixing is continued for 30 s. Lastly, the remaining coarse aggregate, water, and admixture are added, and the entire mixture is mixed and stirred for 60 s. The resulting concrete is then poured to form the desired structure. Concrete specimens of varying heights (100 mm × 100 mm × 100 mm and 100 mm × 100 mm × 400 mm) were prepared based on the mix ratios in Table 2. After 24 h of casting, the concrete specimens were demolded, followed by a subsequent 28-day curing process in a standard curing room maintained at a temperature of 20 ± 2 °C and a relative humidity of ≥95%.

### 2.4. Test Methods and Procedures

#### 2.4.1. Volumetric Weight of Vegetated Lightweight Porous Concrete

Given the substantial differences in composition and structure between vegetative lightweight porous concrete and ordinary concrete, it is anticipated that their bulk densities will also vary significantly. The testing procedure followed the mixed bulk density test used for ordinary concrete, and the concrete was sealed before the test, focusing on Group C concrete with differing porosities to measure the mixed bulk density.

#### 2.4.2. Porosity and Permeability Coefficient

**Figure 2:**
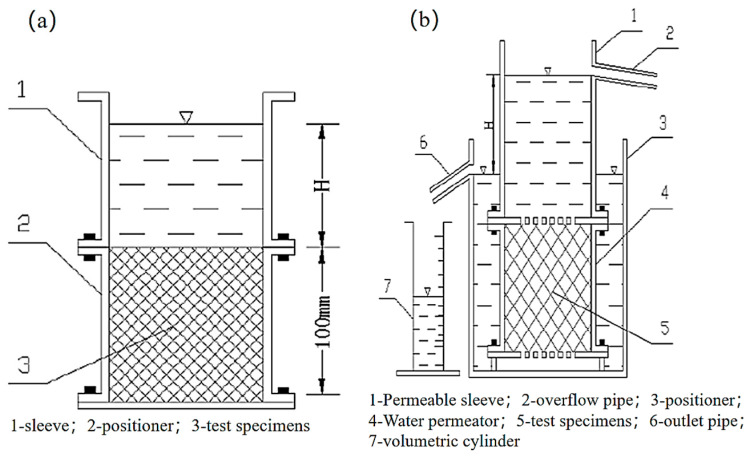
Measuring instrument for porosity and permeability coefficient. (**a**) Porosity measuring instrument; (**b**) permeability coefficient measuring instrument.

The porosity of vegetative lightweight porous concrete (VLPC) was assessed in accordance with the specifications outlined in CJJ/T253-2016 [25]. Simultaneously, the permeability coefficient of VLPC was determined using the constant head method, following the guidelines provided in CJJ/T135-2009 [26], as depicted in Figure 2.

Porosity is determined by measuring the change in water level before and after immersing the sample to ascertain the drainage volume. The formula for calculating the porosity is provided in Equation (5).

(5) $$P=\frac{V-S\times \left(H_{2}-H_{1}\right)}{V}\times 100\%,$$

where *V* denotes the exterior volume of the specimen (cm^3^); *S* denotes the cross-sectional area of the container (cm^2^); *H*_1_ indicates the initial liquid level height (cm); and *H*_2_ indicates the liquid surface height after adding the specimen (cm). When the difference between the maximum value, the minimum value, and the intermediate value exceeds ±0.5%, the sample is re-sampled for the test, and the arithmetic average of the three test results is taken as the final measured value.

The formula for calculating the permeability coefficient is shown in Equation (6):

(6) $$K_{T}=\frac{QD}{AH(t_{2}-t_{1})},$$

where *K* indicates the coefficient of water permeability (cm/s) at water temperature *T* °C; *Q* denotes the amount of water (cm^3^) that passes through the concrete from time *t*_1_ to *t*_2_; *D* denotes the thickness of the macroporous concrete specimen (mm); A denotes the contact area of the macroporous concrete specimen (cm^2^); and H denotes the specified head height (cm). It should be noted that due to the selection of light aggregate with large particle size to prepare VLPC in this test, its water permeability is very good, and the equilibrium head is very small, and it is difficult to reach the specified head. Therefore, the method of balancing the head instead of the specified head is used to calculate the water permeability coefficient.

#### 2.4.3. Mechanical Properties

The strength test for VLPC follows the guidelines specified in GB/T50081-2019 [27]. The testing instrument employed is a 300-ton electro-hydraulic servo-pressure testing machine, with a control loading rate of 0.2 MPa/s. The test ages selected are 7 and 28 days, respectively. As the test blocks used in this study are non-standard specimens, the test flexural strength is adjusted by multiplying it by the conversion coefficient of 0.85, and the test compressive strength is adjusted by multiplying it by the conversion coefficient of 0.95 [27]. The average compressive strength of the three concrete test blocks is considered as the strength value for the specimen group, with the requirement that the deviation between the individual strengths and the average strength should not exceed 15%.

#### 2.4.4. Alkalinity

**Figure 3:**
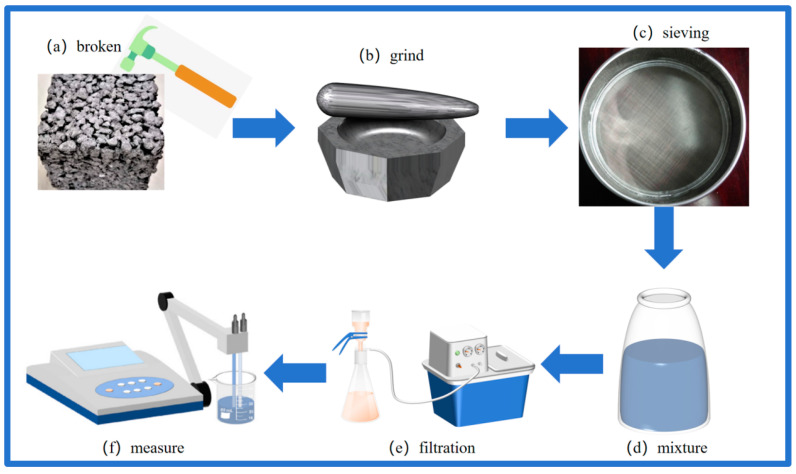
pH Testing. (**a**) Broken sample; (**b**) Grinding sample; (**c**) Sample sieving; (**d**) Mix with distilled water; (**e**) Filter mixture; (**f**) pH measurement.

Due to the inhibitory effect of high alkalinity on seed germination and root growth, the alkalinity of concrete is mitigated by substituting cement types with mineral admixtures [28,29]. The test was conducted following the procedures recommended in IS: 3025 (Part II) -1983, and the sample for the alkalinity test was prepared in accordance with the procedure outlined in ref. [30]. For sample preparation, after reaching the specified age, the sample is extracted from the standard curing chamber and subjected to crushing and grinding, followed by passage through a 0.08 mm sieve. Subsequently, 10 g of the powdered sample is weighed, mixed with 10 times distilled water, and the bottle is sealed to prevent carbonization. After allowing it to sit for 2 h, the mixture is filtered using filter paper. The alkalinity of the concrete is then determined based on the pH value of the filtered solution. The experimental setup for the pH test is illustrated in Figure 3.

## 3. Results

### 3.1. Bulk Weight of Concrete

**Figure 4:**
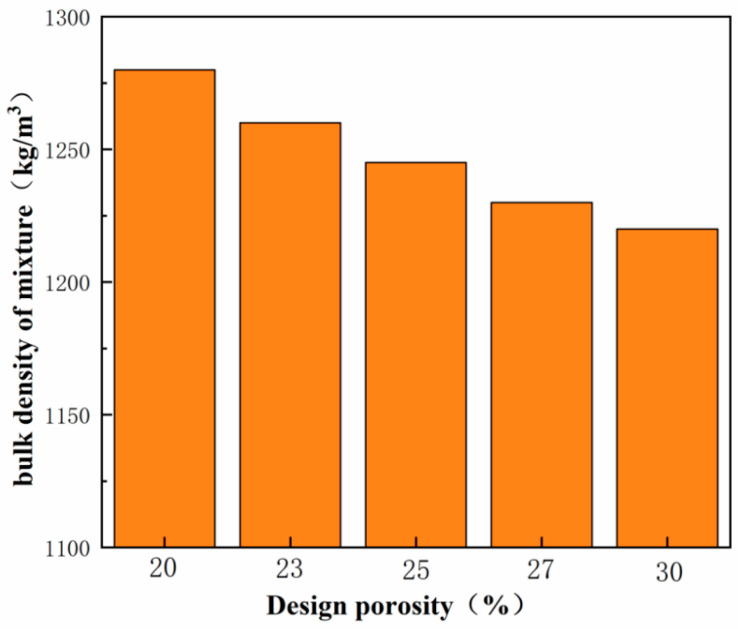
Concrete bulk weight test results.

The results of the volumetric weight determination for mixes with different design porosities are presented in Figure 4. From the figure, it is evident that the bulk weight of fresh VLPC in Group C1 ranges from 1200 kg/m^3^ to 1300 kg/m^3^, approximately half of the bulk weight of ordinary concrete (2200 kg/m^3^ to 2500 kg/m^3^), categorizing VLPC as a lightweight concrete. With the same mixing ratio, an increase in the design porosity correlates with a decrease in the mix weight, demonstrating a predominantly linear relationship between them. In essence, an elevation in the design porosity results in a reduced mass of the active substance within the same volume of concrete, consequently leading to a decline in the mix’s bulk weight.

### 3.2. Porosity and Permeability Coefficient

**Figure 5:**
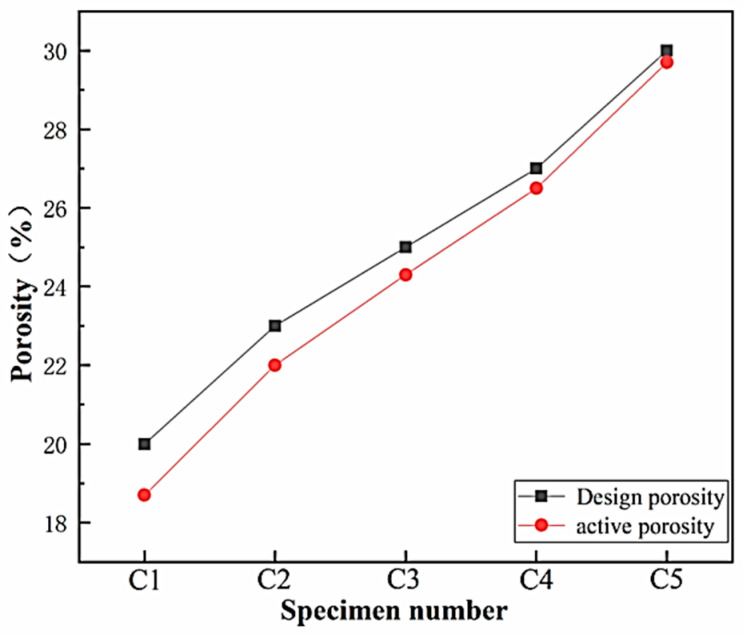
Effective porosity test results.

**Figure 6:**
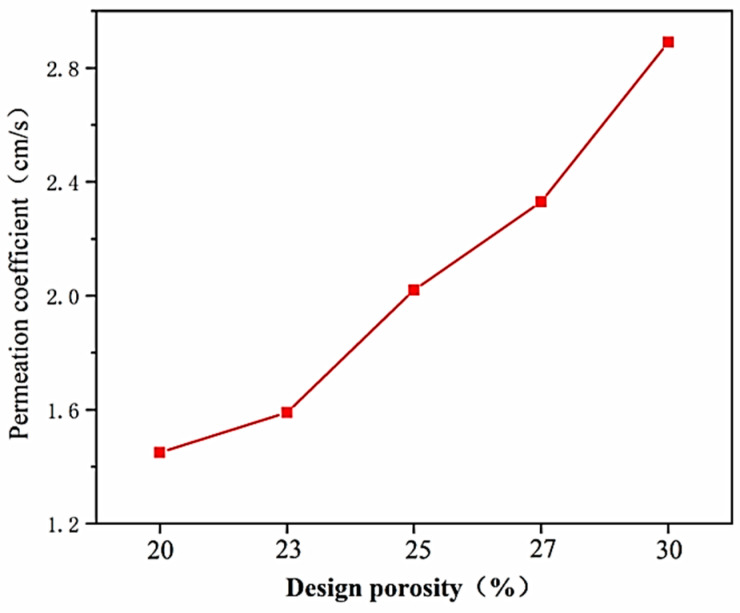
Permeability coefficient test results.

The effective porosity and water permeability coefficient of five test blocks with varying design porosities in Group C were determined. The corresponding relationship between the design porosity and the effective porosity is depicted in Figure 5, while the test results for the water permeability coefficients are illustrated in Figure 6.

From the observations in Figure 5, it is evident that the measured effective porosity of the lightweight porous concrete is smaller than that of the design porosity. This phenomenon is attributed to the occlusion of some pores during the concrete molding process, hindering the formation of continuous or semi-continuous effective porosity. Furthermore, the effective porosity of porous concrete is somewhat influenced by the pressure during molding and the particle size of the coarse aggregate. In this study, a single primary distribution particle size of 10 mm to 20 mm was utilized for the lightweight aggregate, and the disparity between the measured effective porosity and the design porosity was not significant. When the reserved porosity in the design is larger, it is more likely to form continuous or semi-continuous effective porosity, resulting in fewer invalid pores that are completely occluded. Consequently, the larger the design porosity, the closer the measured effective porosity is to the design value. This trend is highly favorable for porous concrete. During the molding process, when the mix of porous concrete is subjected to external forces, the larger reserved pore space can accommodate the movement of the cementitious material slurry wrapped with coarse aggregate. This reduces the formation of completely occluded pores, gradually bringing the measured effective porosity closer to the design porosity.

As depicted in Figure 6, the water permeability coefficient of VLPC increases with the rise in design porosity. This is attributed to the larger design porosity, signifying a smaller amount of cementitious material slurry and a reduced volume of wrapped coarse aggregate surface. As a result, the formation of occluded pores inside the VLPC is less likely, and continuous pores naturally increase, enhancing the water permeability performance.

### 3.3. Mechanical Properties

#### 3.3.1. Effect of Porosity on Mechanical Properties

**Figure 7:**
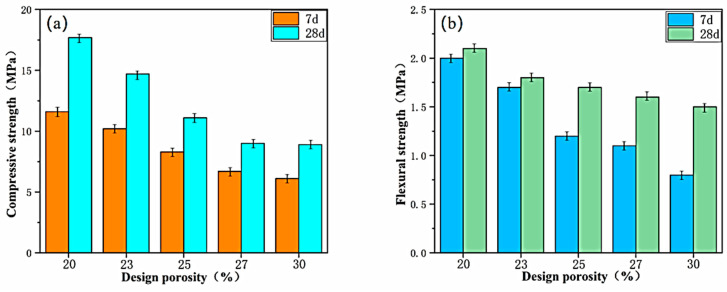
Effect of porosity on compressive and flexural strengths. (**a**) Compressive strength; (**b**) flexural strength.

The compressive and flexural strengths of specimens with five different design porosities in Group C1 are compared, as illustrated in Figure 7. From Figure 7a, it is evident that the cubic compressive strength of porous concrete decreases with increasing design porosity at the same proportion. Notably, at the age of 28 days, the compressive strength of porous concrete with 30% porosity decreases by more than 50% compared to that with 20% porosity. This further confirms the significant impact of the pore structure within the porous concrete on macroscopic strength. For porous concrete intended for engineering applications, it is necessary for its 28-day compressive strength to be no less than 10 MPa and the porosity to be not less than 22%. However, based on the trend observed in the figure, there is very limited scope for improving the compressive strength of porous concrete with an increase in the designed porosity. Therefore, in order to meet the engineering design requirements for strength, the design porosity of porous concrete should not be higher than 30%.

As observed in Figure 7b, the flexural breaking strength of porous concrete decreases with an increase in the porosity. The higher the porosity, the lower the overall compactness of the porous concrete, leading to a reduction in its flexural performance. When the porosity is low, there are relatively fewer pores in the concrete, and the contact area between the particles is larger. This condition enables effective stress transfer, enhancing the flexural capacity of the concrete. Furthermore, lower porosity implies fewer voids within the concrete, reducing internal weak points and promoting a more uniform and robust structure. This aspect contributes to an enhancement in the flexural strength. As the porosity increases, more voids are present in the concrete, some of which may lead to the formation of concentrated stresses and cracks, thereby diminishing the flexural strength of the concrete. In particular, when the connections between the pores are more pronounced, there is a higher likelihood of cracks being transmitted between the pores, further weakening the overall flexural resistance.

#### 3.3.2. Effect of Mineral Admixtures on Mechanical Properties

**Figure 8:**
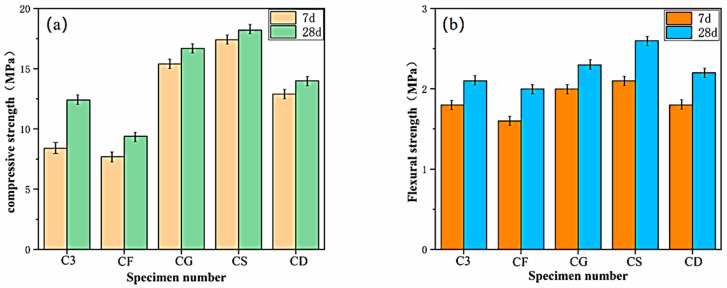
Effect of mineral admixtures on compressive and flexural strengths. (**a**) Compressive strength; (**b**) flexural strength.

The impact of various mineral admixtures on the compressive and flexural strength of VLPC is illustrated in Figure 8. As shown in Figure 8a, both the 7-day and 28-day compressive strengths of VLPC with single doping of GBFS, SF, and double doping of GBFS and FA surpassed that of the C3 group without mineral external dopants. The most notable improvement was observed with single doping of SF, where the 7-day compressive strength increased by 107.1% compared to that of the C3 group. The pozzolanic reaction between Ca(OH)_2_ and SiO_2_ in the SF was expedited by the finer texture of SF. Additionally, the formation of C-S-H resulted from the reaction between the amorphous SiO_2_ in SF and calcium hydroxide from cement hydration, thereby enhancing the compressive strength [31]. Liang Fu et al. discovered that the addition of SF to the cement improved the strength and resistance of the concrete against sulphate attack. These findings align with the results presented in this study [32]. The strength values of the single-doped GBFS increased significantly, and the double-doped group CD also experienced some improvement. However, only the compressive strength values of the single-doped FA decreased, aligning with previous literature reports [33,34]. Ibrahim found that adding FA to concrete reduces the early compressive strength of concrete because FA is less active [35]. The compressive strength values in descending order are SF > GBFA > GBFA + FA > Blank > FA.

This indicates that the addition of mineral admixtures plays a significant role in enhancing the compressive strength of VLPC. Similar to ordinary concrete, the introduction of mineral admixtures improves the uniform distribution of the hydration products in space, accelerating the hydration of cementitious materials through its microcrystalline nucleation effect. Moreover, the unique properties of ultrafine micronized powders, such as slag and silica fume, reduce the amount of water used by cementitious materials. Simultaneously, they refine the pore structure, improve its size and morphology, and enhance the density of the cementitious stone. Consequently, these factors collectively contribute to a substantial increase in the strength performance of porous concrete [36]. The crucial aspect lies in the addition of slag and silica fume, along with other ultra-fine micronized powder. This addition facilitates the reduction of the Ca(OH)_2_ crystal size in the transition zone between cementitious materials and aggregates, weakening the orientation. As a result, the structure of the interface transition layer is significantly improved, enhancing the bond between the cementitious materials and aggregates and inhibiting the destruction of the bond [37].

The impact of mineral external admixtures on the flexural strength of VLPC is illustrated in Figure 8b. The pattern of the effect of mineral external dopants on the flexural strength of VLPC is essentially the same as that observed for the compressive strength. Specifically, the values of the flexural strength are ranked from high to low as SF > GBFA > GBFA + FA > Blank > FA.

### 3.4. Alkalinity

**Figure 9:**
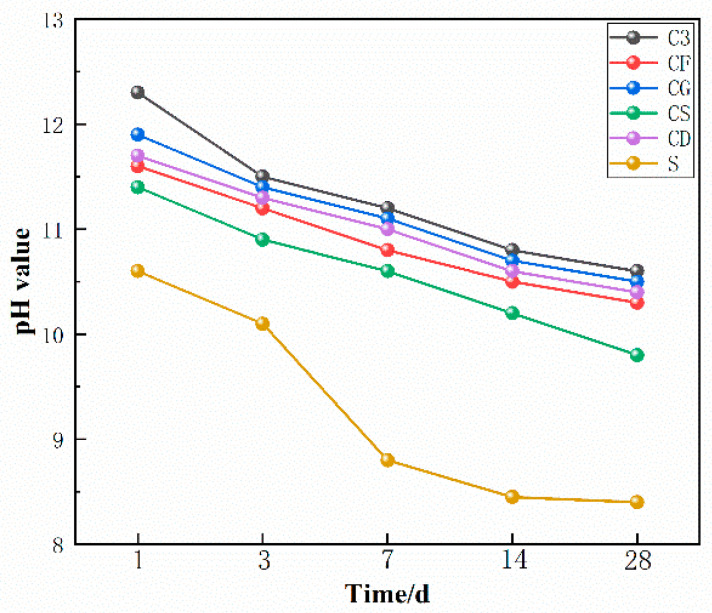
pH value of VLPC at different ages.

The pH of different groups of VLPC was measured, and the test results are depicted in Figure 9. After the hydration reaction of normal silicate cement, various compounds are produced, including C-S-H gel, calcium hydroxide, calcium alumina, and hydrated calcium monosulphoaluminate. The concrete environment is primarily alkaline due to the presence of Ca(OH)_2_, with small amounts of sodium hydroxide and potassium hydroxide also present. Consequently, the Ca(OH)_2_ content of concrete directly influences the alkalinity of its environment [38].

**Figure 10:**
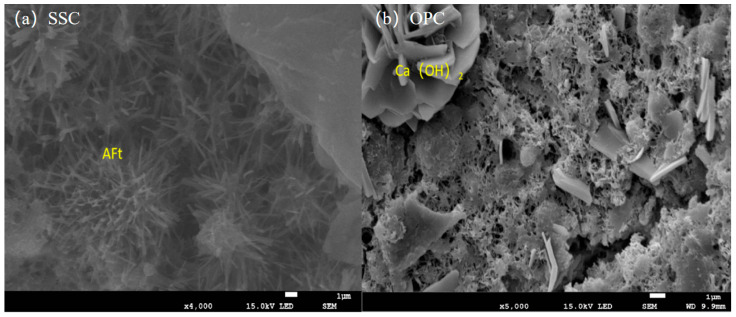
SEM images of SSC hydrated with OPC for 28 days. (**a**) SSC; (**b**) OPC.

Comparing the pH changes of group C3 and group S, the pH of porous concrete prepared with slag sulfoaluminate cement was reduced to less than 8.5 at 28 days, which is 21% lower than that of ordinary silicate cement. This reduction is attributed to the large amount of ettringite generated by the slag sulfoaluminate cement during hydration. The relatively low Ca(OH)_2_ content contributes to the lower alkalinity of the slag sulfoaluminate cement. Figure 10 illustrates the microstructure of the slag sulfoaluminate cement and silicate cement after 14 days of hydration. It can be observed that the slag sulfoaluminate cement contains a significant amount of ettringite, while the ordinary silicate cement generates a large amount of Ca(OH)_2_ crystals.

As the SiO_2_ content in the mineral admixture increases while the CaO content decreases, the water-hardness of SiO_2_ leads it to react with Ca^2+^ to generate new hydrated calcium silicate (C-S-H). This reaction consumes some of the Ca^2+^ ions, thereby reducing the Ca(OH)_2_ content in the concrete. In concrete, Ca(OH)_2_ is the primary alkaline substance that governs the alkalinity of the concrete. Therefore, as the SiO_2_ content increases and the CaO content decreases, the formation of Ca(OH)_2_ in the concrete decreases, consequently lowering the pH of the internal voids of the concrete. Specifically, the CaO content in silica fume, fly ash, and slag powder gradually increases, while the SiO_2_ content gradually decreases. Therefore, the increase in SiO_2_ and the decrease in CaO when using these mineral admixtures result in a reduced amount of Ca(OH)_2_ in the concrete. This ultimately leads to a decrease in the pH of the internal voids of the concrete [39]. This is because the reaction of SiO_2_ with Ca(OH)_2_ reduces the solubility of Ca(OH)_2_, thereby affecting the alkaline environment of concrete. The pH of concrete can be adjusted by controlling the composition and content of mineral admixtures.

The presence of more and larger internal voids in VLPC allows for increased air contact compared to normal concrete. This heightened exposure to air increases the likelihood of carbonation, resulting in a lower pH for porous concrete in comparison to plain concrete. The pH of the internal voids of porous concrete typically decreases to below 10.5 at 28 days. Furthermore, the pH of the internal voids of VLPC decreases further with the incorporation of various mineral admixtures. Among them, silica fume has the most significant effect, reducing its 28-day pH to below 10, sufficiently meeting the growth requirements of some plants. Additionally, other external admixtures can effectively reduce the pH of porous concrete. Based on the degree of pH reduction observed in this study, the order is SF > FA > FA + GBFS > GBFS.

These findings show that the pH value of the interstitials in porous concrete can be effectively reduced by incorporating various mineral admixtures and using low-alkali cement to create a suitable environment for plant growth. In addition, the pH reduction of different admixtures is different, emphasizing the need for careful consideration and reasonable selection of admixtures according to the specific requirements.

## 4. Discussion

VLPC employs lightweight aggregate as the coarse aggregate and lacks fine aggregate, resulting in a density ranging from 1200 kg/m^3^ to 1300 kg/m^3^. This is approximately half of the density of ordinary concrete (2200 kg/m^3^ to 2500 kg/m^3^), enhancing the construction convenience, improving efficiency, and saving materials to a certain extent.

The measured effective porosity of lightweight porous concrete is typically slightly smaller than the design porosity. However, as the design porosity increases, the measured effective porosity gradually approaches the design value. This indicates that the incorporation of larger pre-reserved pores during the molding process assists in forming continuous or semi-continuous effective pores, reducing the occurrence of completely occluded pores. Additionally, it enhances the water permeability of the concrete, which is highly advantageous for porous concrete.

The strength of VLPC decreases with increasing porosity, highlighting the need for a balanced relationship between the porosity and the mechanical properties in the design of porous concrete structures. To ensure sufficient strength, it is essential to control the porosity within an appropriate range, ensuring that the mechanical properties of the concrete meet the design requirements.

The incorporation of mineral admixtures is crucial for enhancing the strength of VLPC. Both slag powder and silica fume contribute significantly to early and mid-late strength development. In cases where economic considerations come into play, a double mix with an appropriate proportion of fly ash, replacing slag powder, can be employed to achieve strength improvement. However, it is not advisable to use fly ash alone. This is because, given that the early strength of vegetative lightweight porous concrete is not inherently high, the addition of fly ash could further reduce its early strength, presenting challenges in the construction of vegetative lightweight porous concrete.

The inclusion of mineral admixtures can variably decrease the alkalinity of VLPC. Higher SiO_2_ content in minerals leads to reduced Ca(OH)_2_ generation, resulting in a lower pH value. When SSC is utilized, the alkalinity of VLPC is notably diminished compared to ordinary concrete, primarily due to its main hydration product being dominated by calcium alumina with a limited Ca(OH)_2_ content.

## 5. Limitations and Prospects

### 5.1. Limitations

In our study, we employed a constrained set of materials, pore size designs, and mineral additives to fabricate VLPC. However, this restricted selection may not fully encompass the potential variations and combinations that could optimize the overall performance of VLPC. Subsequent research and experimentation involving a broader range of materials are necessary to comprehensively explore and enhance its properties.

Our experiments were carried out in controlled laboratory conditions, neglecting the potential impact of long-term exposure to environmental factors or aging. In real-world applications, VLPC may face various weathering conditions, including temperature fluctuations, moisture, and chemical exposure, which could influence its performance and durability. Future studies should include field tests or accelerated aging experiments to evaluate the long-term behavior of VLPC.

### 5.2. Prospects

VLPC holds significant potential for diverse applications. It proves beneficial in road and pavement construction, green roofs, retaining walls, and landscaping projects. The porous structure of VLPC facilitates water infiltration, addressing urban stormwater runoff issues, and contributing to the enhanced ecological balance of urban environments.

Future research can concentrate on broadening the spectrum of cementitious materials and mineral additives employed in VLPC production. Through the exploration of diverse combinations, researchers aim to enhance the mechanical properties, improve durability, and reduce the costs associated with the manufacturing process.

While VLPC exhibits promise, further studies and practical applications are essential to validate its performance in diverse environments and construction scenarios. Collaborative efforts between researchers, engineers, and construction professionals will be crucial to unlock the full potential of VLPC and to overcome the existing limitations.

## Figures and Tables

**Table 1:** Chemical composition of SSC, OPC, FA, SF and GBFS.

Oxides%	CaO	SiO_2_	Al_2_O_3_	Fe_2_O_3_	MgO	SO_3_	TiO_2_	LOI
P.II 52. 5	64.50	22.04	4.76	3.10	0.92	1.90	/	1.01
SSC52.5	39.25	26.95	13.21	0.41	8.51	7.84	0.53	0.31
FA	3.36	57.74	27.08	6.34	1.11	0.18	/	3.61
SF	0.4	91.05	1.73	0.91	0.78	0.27	/	1.02
GBFS	37.19	31.77	15.36	0.63	10.15	1.16	/	1.02

**Table 2:** Mix proportion (kg/m^3^).

Number	Design Porosity (%)	Cement	Aggregate	Water	FA	GBFS	SF	JM-2	Water-Cement Ratio
C1	20	406	524	105.6				2.18	0.26
C2	23	356	524	92.6				1.79	0.26
C3	25	322	524	83.7				1.73	0.26
C4	27	288	524	74.9				1.42	0.26
C5	30	234	524	60.8				1.05	0.26
CF	25	170	513	79.2	113.0			1.42	0.28
CG	25	182	513	88.2		122.0		1.52	0.29
CS	25	325	513	79.7			6.6	1.66	0.24
CD	25	177	513	82.6	59.0	59.0		1.48	0.28
S	25	335	524	83.7				1.73	0.26

Note: C stands for common silicate cement; S stands for slag sulfur–aluminate cement. CF, CG, CS, and CD represent samples with single-doped FA, single-doped GBFS, single-doped SF, and double-doped FA and GBFS, respectively.

## Data Availability

Data are contained within the article.
